# Using the Habit App for Weight Loss Problem Solving: Development and Feasibility Study

**DOI:** 10.2196/mhealth.9801

**Published:** 2018-06-20

**Authors:** Sherry Pagoto, Bengisu Tulu, Emmanuel Agu, Molly E Waring, Jessica L Oleski, Danielle E Jake-Schoffman

**Affiliations:** ^1^ Institute for Collaboration on Health, Intervention, and Policy Department of Allied Health Sciences University of Connecticut Storrs, CT United States; ^2^ Foisie Business School Worcester Polytechnic Institute Worcester, MA United States; ^3^ Computer Science Department Worcester Polytechnic Institute Worcester, MA United States; ^4^ Preventive and Behavioral Medicine Department of Medicine University of Massachusetts Medical School Worcester, MA United States

**Keywords:** mobile app, mHealth, weight loss, obesity, problem solving

## Abstract

**Background:**

Reviews of weight loss mobile apps have revealed they include very few evidence-based features, relying mostly on self-monitoring. Unfortunately, adherence to self-monitoring is often low, especially among patients with motivational challenges. One behavioral strategy that is leveraged in virtually every visit of behavioral weight loss interventions and is specifically used to deal with adherence and motivational issues is problem solving. Problem solving has been successfully implemented in depression mobile apps, but not yet in weight loss apps.

**Objective:**

This study describes the development and feasibility testing of the Habit app, which was designed to automate problem-solving therapy for weight loss.

**Methods:**

Two iterative single-arm pilot studies were conducted to evaluate the feasibility and acceptability of the Habit app. In each pilot study, adults who were overweight or obese were enrolled in an 8-week intervention that included the Habit app plus support via a private Facebook group. Feasibility outcomes included retention, app usage, usability, and acceptability. Changes in problem-solving skills and weight over 8 weeks are described, as well as app usage and weight change at 16 weeks.

**Results:**

Results from both pilots show acceptable use of the Habit app over 8 weeks with on average two to three uses per week, the recommended rate of use. Acceptability ratings were mixed such that 54% (13/24) and 73% (11/15) of participants found the diet solutions helpful and 71% (17/24) and 80% (12/15) found setting reminders for habits helpful in pilots 1 and 2, respectively. In both pilots, participants lost significant weight (*P*=.005 and *P*=.03, respectively). In neither pilot was an effect on problem-solving skills observed (*P*=.62 and *P*=.27, respectively).

**Conclusions:**

Problem-solving therapy for weight loss is feasible to implement in a mobile app environment; however, automated delivery may not impact problem-solving skills as has been observed previously via human delivery.

**Trial Registration:**

ClinicalTrials.gov NCT02192905; https://clinicaltrials.gov/ct2/show/NCT02192905 (Archived by WebCite at http://www.webcitation.org/6zPQmvOF2)

## Introduction

Reviews of weight loss mobile apps have revealed they include very few evidence-based features [1,2], rarely involve behavioral experts in the developmental process [1,3], and lack efficacy data [1]. The most common features in weight loss apps are self-monitoring and goal setting, which is a narrow list given that evidence-based behavioral weight loss programs deliver up to 20 different behavioral strategies [2]. Further, a behavior change taxonomy for diet and exercise includes 40 behavioral strategies [4]. Science that demonstrates how specific behavioral strategies can be effectively implemented via a mobile platform could improve the impact of weight loss apps.

Reliance on self-monitoring is a problem because adherence to self-monitoring is often low. In one study that prescribed a commercial weight loss mobile app to 212 primary care patients, more than half (56%) did not use it in the first month and by 6 months 84% were not using it [5]. With self-monitoring being the cornerstone feature, impact is limited to those willing to self-monitor regularly. A comprehensive set of behavioral strategies is needed in weight loss apps to increase relevance, utility, and impact in people at varying levels of adherence and motivation.

Some apps include a more comprehensive suite of behavioral strategies. For example, the Noom Coach app [6] connects users to a live coach who is trained to deliver the Diabetes Prevention Program, a behavioral weight loss program that includes 20 behavioral strategies [7]. The app itself does not deliver the strategies, but rather a trained coach, which costs US $45 to $90 per month. Higher levels of sophistication in behavioral strategies generally come with a cost relative to other weight loss apps, the majority of which are free. To the extent that an app can be programmed to deliver additional behavioral strategies automatically, less coach time and expense may be required which would facilitate wider reach and impact.

One behavioral strategy that is leveraged in virtually every visit of behavioral weight loss interventions and is specifically used to deal with adherence and motivational issues is problem solving. Problem solving is a counseling technique used to help an individual identify barriers to behavior change and generate solutions to be iteratively attempted until barriers are overcome [8]. In practice, the counselor works through five steps with the patient, including (1) identifying a significant barrier to behavior change, (2) brainstorming a list of solutions with the patient, (3) having the patient select a solution he/she would be willing to try over the next week, (4) scheduling a time to attempt the solution, and (5) evaluating the outcome and trying additional solutions until the problem is solved. At the end of each session of behavioral weight loss treatment, patients are asked to identify barriers that are likely to arise as they attempt the homework assignment and they are then assisted in making a plan to overcome those barriers [9]. Additionally, an entire session is devoted to problem-solving skills as well so that patients get more intensive training in how to make progress in the presence of barriers [7]. Given the systematic process of problem solving, it would seem conducive to being facilitated via a mobile app.

Another advantage of a problem-solving app is that studies have established problem solving is an “active ingredient” of behavioral weight loss interventions [10,11]. Problem solving has been shown to be effective as a standalone intervention for weight loss maintenance [12] and is a strong predictor of weight loss outcomes [13]. Finally, a problem-solving app can be designed to address a wide range of weight loss barriers.

Although problem solving has not been incorporated in a weight loss mobile app, it is a staple in multistrategy depression mobile apps [14-16] and three studies tested technology-based programs for depression exclusively focused on problem solving [17-19]. Two studies of Web-based problem-solving depression programs revealed statistically and clinically significant improvements in depression relative to waitlist controls [17,18], although one was only effective when paired with email coaching [18]. In a recent remote trial of a problem-solving therapy app for depression, participants were emailed links to a problem-solving app but they were provided no human contact during the study. Less than half of participants downloaded the app, which suggests that human contact may be necessary at treatment initiation [19]. Nonetheless, participants with elevated depression scores in the problem-solving app condition showed greater declines in depression relative to those in a control app condition. The lack of human contact may have undermined outcomes, given the far higher download rates in studies providing human contact [16]. Studies of weight loss mobile apps failed to establish their efficacy in the absence of other support [5]. As such, the problem-solving app proposed in this study will be paired with access to a social media-delivered weight loss intervention, which has been shown to have modest effects on weight [20-22].

This study describes the development and feasibility testing of the Habit app, which was designed to automate the problem-solving process for common weight loss barriers. Once developed, two iterative pilot studies were performed to evaluate the feasibility and acceptability of the Habit app. In each pilot study, adults who were overweight or obese were enrolled in an 8-week intervention that included the Habit app plus support via a private Facebook group. Feasibility outcomes include retention, app usage, usability, and acceptability. Changes in problem-solving skills and weight over 8 weeks are described, as well as app usage and weight loss at 16 weeks. After pilot 1, refinements were made to the program.

## Methods

### Habit App Development: Overview

To develop a database of weight loss problems, a steering committee of clinicians was queried and problem-solving sessions with patients were conducted. Solutions were derived in problem-solving sessions and by our investigative team, who have extensive experience in behavioral weight loss counseling. An algorithm was then designed to ensure solutions provided by the app were tailored to user characteristics.

#### Steering Committee of Clinicians

Eleven counselors (4 dietitians, 5 psychologists, 1 Master’s-level counselor, and 1 health educator) with experience counseling patients for weight management and practicing at UMass Memorial Medical Center composed our steering committee and were asked to name the most common problems patients experience when it comes to diet and exercise. A total of 77 problems were identified.

#### Problem-Solving Sessions

Adults with obesity (N=30; female: 27/30, 90%; age: mean 47, SD 13 years; body mass index [BMI]: mean 35.9, SD 4.2 kg/m^2^; non-Hispanic white: 23/30, 77%) were recruited via ads to participate in a single session of problem solving with a weight loss counselor. Adults were eligible if they had BMI between 30 and 45 kg/m^2^ and were currently trying to lose weight. Each participant attended a 1-hour session with a weight loss counselor in which the problem-solving session of the Diabetes Prevention Protocol Lifestyle Intervention was administered. These sessions followed the five-step problem-solving process described previously. Each participant was asked to discuss one diet and one exercise problem, and the counselor cycled through the process for each and came up with 10 solutions for each problem. These sessions generated 60 problems and 600 solutions, although many were duplicates.

#### Categorization of Problems

A total of 137 responses for problems (77 from steering committee, 60 from patients) were reviewed by the investigative team who removed duplicates and infrequent responses and classified the remaining into nine diet and six exercise problem categories (see Table 1).

#### Algorithm Development

**Figure 1:**
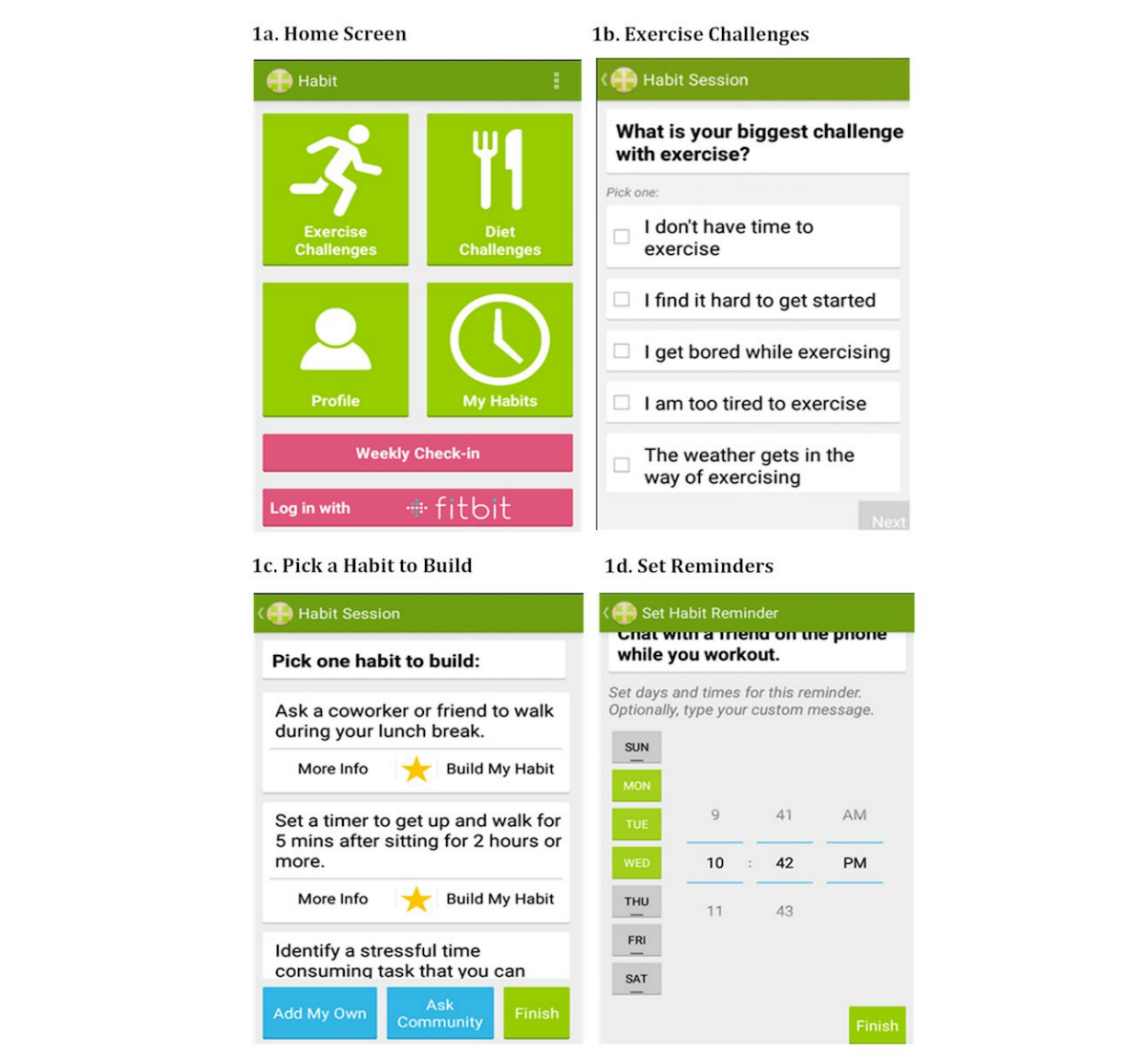
Habit app screenshots.

Many of the solutions generated were specific to user characteristics (eg, stay-at-home mom) and/or lifestyle factors (eg, currently exercises three times per week). For example, among patients who said they eat when they are bored, some engaged in this habit in the evening after work, whereas others while taking care of children at home during the day. Solutions would be different for these scenarios. To the extent the app provides many irrelevant solutions, the user would be unnecessarily burdened. As such, each problem was accompanied by a set of one to five questions regarding user and lifestyle characteristics that would eliminate as many irrelevant solutions for the user as possible. The user and lifestyle characteristics that had relevance to multiple problems were queried during the profile setup (see Figure 1). User characteristics included, but were not limited to, employment status, parental status, medical conditions, and climate. Lifestyle characteristics included, but were not limited to, sleep and work hours, current exercise regimen, and exercise preferences.

**Table 1:** Problem categories for diet and exercise.

Categories		Illustrative examples	Example solutions
**Diet**			
	Stress	“I eat too much when I’m stressed”	“Make a list of stress foods and make sure not to bring them into the house”
	Willpower	“I can’t resist junk food people bring to work”	“Bring healthy snacks to work to eat instead”
	Hunger	“I feel hungry from 3 pm until bedtime”	“Have cut up fruit and veggies in the fridge ready to snack on”
	Eat when bored	“I snack a lot when I’m home with the kids”	“Make a list of activities that involve going places that do not have food (eg, library)”
	Restaurants	“It’s too hard to track what I eat when I eat out”	“Reduce the number of meals you eat out by 1 per week”
	Weekends	“The lack of structure on weekends makes it harder to watch diet”	“Plan menus on the weekend like you would on a weekday”
	Parties/holidays	“I want to try all the foods at a party”	“Take some food home to eat at your next meal time”
	Sugary beverages	“I drink too much soda”	“Switch some sodas for noncalorie seltzers”
	Alcohol	“I end up eating more when I drink”	“Have a glass of water between alcoholic beverages to slow you down and fill you up”
**Exercise**			
	Time	“It’s hard to make time to exercise”	“Schedule in exercise like you would other appointments”
	Hard to get started	“I’m so unmotivated to exercise”	“Start with a small goal like 10 minutes of exercise per day”
	Boring	“I find exercise boring”	“Do something enjoyable while exercising like watching TV or listening to a book”
	Too tired	“I’m too tired to exercise”	“Try these sleep hygiene techniques to improve your sleep”
	Weather	“I miss workouts due to bad weather”	“Develop an indoor exercise plan to use as a backup”
	Pain/injury	“Knee pain prevents me from exercising”	“Make appointment with a physical therapist to learn exercises that won’t cause pain”

### Habit App

#### Home Screen

The home screen of the Habit app (see Figure 1) gives users the choice to update their profile, address a diet or exercise challenge (“problem solving”), view solutions they currently have scheduled, or complete a weekly check-in. In usability testing, participants did not prefer the terms “problems,” “barriers,” or “solutions,” but rather suggested “challenges” and “habits.” Therefore, language according to their preferences was adopted. Each feature is described subsequently.

#### Profile

The Habit app prompts users to set up a profile in which they enter their height, weight, goals, current exercise habits and preferences, employment status, parental status, and notification preferences. Users can indicate how often they prefer to be reminded to weigh in. In our pilot studies, users were instructed to weigh in weekly and work on one to two habits per week. Fitbit users can log in using their Fitbit credentials and transfer data logged by the Fitbit device or entered in the Fitbit app, which allowed Habit to remind the user to problem solve if they exceeded their calorie goal. The Habit profile page includes a weight graph and lists the participant’s current list of habits.

#### Problem Solving

The first step of problem solving is “identify the problem.” For this step, users can choose a diet or exercise challenge. Once selected, a list of challenges appears (see Figure 1). Once they choose a challenge, they are asked to further specify the challenge by answering one to five questions, depending on the challenge selected. Participants also have the option of adding habits to the app if the habit they would like to work on is not included in the app’s database. The second step of problem solving is “brainstorm solutions.” A screen appears with a list of solutions, referred to as “habits” in the app (see Figure 1). For each, the user can click “more info” to be linked to an online article that elaborates on the importance of the habit and how to implement it. The third step of problem solving is “pick a solution to try.” For this, the user selects “build my habit” for the habit they want to try. The fourth step of problem solving is “make a plan.” A list of days of the week and hours of the day is presented and the app prompts the user to set reminders to implement the habit (see Figure 1). A reminder notification occurs on the selected time and days until the user deactivates the notification. All scheduled habits are viewable by clicking the My Habits button on the main page. Users can delete the reminders here and view all current habits and all habits in their history. The fifth step of problem solving is “follow-up.” Each week, the user will be prompted to complete a weekly check-in, which asks the user to enter their current weight, to indicate all scheduled habits they successfully accomplished for the week, to indicate all the habits they would like to work on for the coming week, and to select new habits. Those indicating a desire to select new habits are brought back to the main page to enter a challenge.

### Overview of Pilot Series

Two sequential single-arm pilot studies were conducted to examine the feasibility, acceptability, and use of the Habit app. Changes in problem-solving skills and weight over 8 weeks were described. The Habit app was paired with a private counselor-led Facebook group. Identical recruitment, screening, inclusion and exclusion criteria, and measures were used for pilots 1 and 2. Intervention refinements were made for pilot 2 based on pilot 1 results. All work was approved by the University of Massachusetts Medical School Institutional Review Board.

#### Recruitment and Screening

Participants were recruited for pilot 1 in December 2015 through January 2016, and for pilot 2 in July 2016 through September 2016. Online recruitment was used with ads posted in Facebook groups and Craigslist pages throughout the United States. A link to an initial online survey was included in the recruitment ad, which directed participants to a study description and initial screening questions. Eligible individuals were sent a consent form and booked for a telephone screening call. During the call, staff reviewed the consent document, asked remaining eligibility-related questions, and emailed a link to the baseline survey. The survey included questions about participant characteristics and problem-solving skills. Eligible participants were sent a unique link to download the app and join the Facebook intervention group and sent a Wi-Fi scale to measure their body weight during the study.

#### Inclusion and Exclusion Criteria

Participants were required to be age 18 years or older, have a BMI between 30 and 45 kg/m^2^, currently use an Android smartphone daily, have a Gmail account or be willing to open one (the app was located on Google Play), have connectivity to the internet at home and work, and have written physician clearance. Participants were excluded if they were not regular users of Facebook, were not comfortable using a weight loss app, had severe mental illness or substance abuse, were pregnant or lactating, had bariatric surgery, were taking medication that affects weight, had type 1 or 2 diabetes, or had a medical condition that precludes lifestyle changes.

#### Habit App Orientation

Participants were emailed orientation materials and setup instructions for the app and Wi-Fi scale. The app and Facebook group were explained, including guidance on participation (eg, work on one to two challenges per week, check Facebook page daily).

#### Intervention

Participants were asked to use the Habit app to work on one to two weight loss challenges each week for 8 weeks while participating in a private counselor-led Facebook group. The counselor posted once per day in the Facebook group. On Monday mornings, the counselor’s post prompted participants to use the Habit app to select one to two habits for the week, and on Sunday mornings they were asked to report how they did with the habits they chose for that week. Posts on Tuesday through Saturday covered basic behavioral weight loss strategies, including nutrition education, developing a physical activity regimen, stress management, negative thinking, and others as described elsewhere [23]. Content was consistent with that covered in the Diabetes Prevention Program Lifestyle intervention [7].

#### Follow-Up Assessments

Participants completed weigh-in and a follow-up survey at 8 weeks. At 16 weeks, their weight and use of the app over the previous 8 weeks was queried. Compensation of US $40 was provided at the end of the study.

### Measures

#### Retention

Retention was defined as the percentage of participants who completed the follow-up weigh-in (via Wi-Fi scale) at 8 weeks.

#### App Usage

Participants received a weekly survey that asked how many times they used the Habit app over the past week, how many habits they tried, and how many reminder notifications they scheduled. At 16 weeks, they were asked to estimate how many times in the past 8 weeks (since the intervention ended) they used the app.

#### System Usability Scale

The System Usability Scale (SUS) includes 10 Likert scale items to estimate overall usability and participant satisfaction and is useful in comparing different versions of the same system [24]. A SUS score greater than 70 is considered adequate and a score greater than 80 is high [24,25].

#### Facebook Group Engagement

Engagement in the Facebook group was defined as the total number of likes, comments, and original posts. Engagement data was captured via the Facebook API using a computer program.

#### Acceptability

At the end of each pilot, participants were asked to rate on a five-point Likert scale how much they agreed with the following statements: “The diet-related habits in the Habit app were helpful for me,” “The exercise-related habits in the Habit app were helpful for me,” “Being able to set reminders was helpful to me,” “Coach posts on Facebook were helpful,” “Participants posts on Facebook were helpful,” and “I would recommend Habit app to my friends and family.” Participants were also asked to list any challenges they experienced that were not addressed by Habit app.

#### Social Problem-Solving Inventory-Revised

The Social Problem-Solving Inventory-Revised (SPSI-R), which measures an individual’s problem-solving ability, was administered at baseline and at 8 weeks. Scores are sensitive to change in interventions of problem-solving therapies [26,27] and have construct validity [28].

#### Weight

Weight was obtained using the Fitbit Aria scale at baseline, 8 weeks, 16 weeks, and whenever participants weighed themselves during the intervention period. Participants were mailed a scale once they were determined to be eligible and provided staff with log-in information for the scale so they could record weight during assessments. At the end of the study, participants were allowed to keep the scale.

### Analytic Plan

In both pilots, retention, app usage, Facebook group engagement, and acceptability were summarized. Paired-samples *t* tests were used to evaluate change in problem solving and weight over 8 weeks. Baseline value carried forward was used to impute missing data at 8 weeks for problem solving and weight.

### Sample Size Considerations

Leon et al [29] stated, “Power analyses should not be presented in a pilot study that does not propose inferential results.” As they and others recommend [29,30], we based the sample size on necessities for examining feasibility. For each pilot, our recruitment target was a sample size of 20, which would allow us to identify usability issues under conditions of regular use. In pilot 1, we exceeded this target (N=27) so we recruited somewhat less in pilot 2 (N=16) for a total sample of 43. This sample size is consistent with recent pilot studies of similar technology-delivered weight loss interventions [23,31].

## Results

### Pilot 1

#### Participant Characteristics

Participants (N=27) had a mean age of 37.22 (SD 11.55) years and a mean baseline BMI of 31.14 (SD 4.63) kg/m^2^; 67% (18/27) were female and 85% (23/27) were non-Hispanic white (see Table 2).

#### Feasibility

Three of 27 participants (11%) did not provide weight data at 8 weeks. Participants reported using the Habit app on average a total of 22.96 (SD 18.77) times over 8 weeks, with 18 of 27 participants (67%) using the app during week 8 (see Table 3). Participants reported trying a mean 2.46 (SD 1.61) habits per week and scheduled reminders for a mean 2.37 (SD 1.78) habits per week. Participants added a mean 3.59 (SD 6.56, range 0-29) habits of their own to the app. At 16 weeks, 15 participants (59%) had used the app at least once and the mean number of uses was 7.27 (SD 9.86). The mean SUS score was acceptable (mean 73.00, SD 15.82).

Participants engaged with the Facebook group a mean 70.96 (SD 77.21; range 0-265) times. Only one participant (4%) did not engage at all in the Facebook group. “Likes” were the most common form of engagement with a mean 41.44 (SD 58.42), followed by comments (mean 24.22, SD 26.81). Original posts were less frequent with a mean 5.30 (SD 6.24) total per participant. Participants made a mean 8.87 (SD 9.65) engagements per week.

In terms of acceptability, of the 24 (89%) who completed the 8-week follow-up survey, 54% (13/24) agreed/strongly agreed the diet habits in the app were helpful, and 14 of 24 (58%) agreed/strongly agreed the exercise habits in the app were helpful. Most (17/24, 71%) agreed/strongly agreed that being able to set reminders was helpful and most agreed/strongly agreed that the coach posts in the Facebook group were helpful (20/24, 83%), but somewhat fewer agreed/strongly agreed (15/24, 63%) that participants’ posts in the Facebook group were helpful. Thirteen (54%) agreed or strongly agreed that they would recommend the Habit app to friends. Three participants mentioned a total of three problems that were not addressed in the app.

#### Problem Solving and Weight Loss

No significant changes were observed in total problem-solving score (*t*_26_=–0.50, *P*=.62) from baseline to 8 weeks.

Participants’ weight changed by a mean –3.53 (SD 5.91, range –20.10 to 6.80) pounds (*t*_26_=3.10, *P*=.005), which is –1.61% of baseline weight (SD 2.52, range –7 to 3), or 0.20% (SD 0.03%) per week (see Table 4). At 16 weeks, participants’ weight changed by a mean –3.33 (SD 10.12, range –28.90 to 12.60) pounds from baseline (*t*_26_=1.71, *P*=.09), which is –1.26% of baseline weight (SD 4.55, range –12% to 9%).

**Table 2:** Participant characteristics in pilots 1 and 2.

Demographics	Pilot 1 (N=27)	Pilot 2 (N=16)
Gender (female), n (%)	18 (67)	12 (75)
Age (years), mean (SD)	37.22 (11.55)	37.35 (10.85)
Race/ethnicity (white), n (%)	23 (85)	12 (75)
Baseline body mass index (kg/m^2^), mean (SD)	31.15 (4.63)	32.96 (5.99)

**Table 3:** App usage and system usability for pilots 1 and 2.

Use and usability	Pilot 1 (N=27)	Pilot 2 (N=16)
App usage during 8 weeks of intervention, mean (SD)	22.9 (18.7)	24.9 (19.8)
App usage during 8 weeks following intervention, mean (SD)	7.27 (9.86)	3.73 (6.43)
Participants using app during 8 weeks following intervention, n (%)	16 (59)	8 (50)
System Usability Scale score, mean (SD)	73.00 (15.82)	64.00 (11.83)

**Table 4:** Weight change and problem-solving skills in pilots 1 and 2.

Weight change and problem solving skills	Pilot 1 (N=27)	Pilot 2 (N=16)
Percent weight loss at 8 weeks (%), mean (SD)	–1.61 (2.62)^a^	–2.25 (3.92)^a^
Participants losing ≥3% at 8 weeks, n (%)	10 (37)	8 (50)
Percent weight loss at 16 weeks (%), mean (SD)	–1.26 (4.55)	–1.03 (5.31)
Participants losing ≥3% at 16 weeks, n (%)	7 (26)	9 (56)
SPSI-R^b^ total standard score change, mean (SD)	0.67 (6.83)	–2.68 (9.32)

^a^*P*<.05.

^b^SPSI-R: Social Problem-Solving Inventory-Revised.

### Modifications

Following pilot 1, findings were reviewed by the investigative team to determine changes needed to the Habit app and/or the intervention model. Participants running older versions of Android on their phones had a disproportionate amount of bugs, thus the Android version was restricted to version 4.0 (released October 2011) or later for pilot 2. Further, given that no improvement was observed in problem-solving skills, the team decided to add a webinar that demonstrated the problem-solving process to participants before using the app. Simply using the app might not result in learning the problem-solving process, but this added tutorial might give participants a clear understanding of the problem-solving process facilitated by the app and how they might generate habits to add to the app or solve problems even without the app. Before receiving the intervention, participants attended the problem-solving webinar led by the principal investigator. In the webinar, participants learned that the Habit app was designed to deliver a five-step problem-solving process known to be effective in helping people change behavior. They received a rationale for problem solving and the process was modeled with a volunteer from the group who shared a problem. The investigator engaged the group in brainstorming and the volunteer was asked to select a solution and make a plan to try it. Participants were encouraged to share problems in the Facebook group to tap the group for brainstorming, particularly if problems arose that were not addressed in the Habit app.

### Pilot 2

#### Participant Characteristics

Participants (N=16) had a mean age of 37.35 (SD 10.85) years and a mean baseline BMI of 32.96 (SD 5.99) kg/m^2^; 71% (11/16) were female and 71% (11/16) were non-Hispanic white (see Table 2).

#### Feasibility

All participants (16/16; 100% retention) provided weight data at 8 weeks. Participants reported using the Habit app a mean 24.93 (SD 19.86) times over 8 weeks, with 9 of 16 participants (56%) using the app on week 8 (see Table 3). They reported trying a mean 1.67 (SD 0.98) habits per week and scheduled reminders for a mean 2.11 (SD 1.60) habits per week. Participants added a mean 2.93 (SD 3.66, range 0-12) habits of their own to the app. At 16 weeks, 50% (8/16) of participants had used the app at least once and mean number of uses was 3.73 (SD 6.43). Mean SUS scores were below the acceptable cut-off of 70 (mean 64.00, SD 11.83).

Mean total engagement was 40.25 (SD 12.94; range 1-97). “Likes” were the most common form of engagement with a mean total of 18.50 (SD 20.32), followed by comments (mean 21.00, SD 12.94). Original posts were less frequent with only a mean 0.75 (SD 1.24) per participant. Participants made a mean 5.03 (SD 3.72) engagements per week.

All but one participant completed the 8-week follow-up survey on acceptability (see Table 5). Of the 15 who did, nearly three-quarters (11/15, 73%) agreed/strongly agreed that the diet habits in the app were helpful, and 10 of 15 (67%) agreed/strongly agreed the exercise habits were helpful. Most participants agreed/strongly agreed that being able to set up reminders and the coach posts were helpful (12/15, 80% and 13/15, 87%, respectively). Most (12/15, 80%) also agreed/strongly agreed that participants’ posts were helpful. Five participants (33%) said they would recommend the Habit app to a friend. Four participants mentioned a total of four problems that were not addressed in the app.

**Table 5:** Acceptability of the Habit app in pilots 1 and 2.

Acceptability	Pilot 1 (N=24)^a^, n (%)	Pilot 2 (N=15)^a^, n (%)
Diet solutions were helpful (% agree or strongly agree)	13 (54)	11 (73)
Exercise solutions were helpful (% agree or strongly agree)	14 (58)	10 (67)
Being able to set reminders was helpful (% agree or strongly agree)	17 (70)	12 (80)
Facebook: coach posts were helpful (% agree or strongly agree)	20 (83)	13 (87)
Facebook: participants posts were helpful (% agree or strongly agree)	15 (63)	12 (80)
Would recommend Habit app to friends/family (% agree or strongly agree)	13 (54)	5 (33)

^a^Three participants did not complete the survey in pilot 1; one did not complete the survey in pilot 2.

#### Problem Solving and Weight Loss

No significant changes were observed in total problem-solving score (*t*_15_=1.15, *P*=.27). Participants weight changed by a mean –5.01 pounds (SD 8.04, range –24.10 to 8.10), which was 2.25% of baseline weight (SD 3.92, range –8 to 6; *t*_15_=2.49, *P*=.03) or 0.28% (SD 0.05) per week of baseline weight. At 16 weeks, participants weight changed by a mean –2.37 pounds (SD 10.68, range –22.90 to 16.20; *t*_15_=0.89, *P*=.39), which is on average –1.03% of baseline weight (SD 5.31, range –12 to 8).

## Discussion

Results from pilots 1 and 2 show acceptable use of the Habit app over 8 weeks with, on average, two to three uses per week, which was the rate of use recommended in the program. Acceptability ratings were mixed such that 54% (pilot 1) to 73% (pilot 2) of participants found the diet and/or exercise solutions helpful and the majority (pilot 1: 70%; pilot 2: 80%) found setting reminders for habits helpful, but only 54% (pilot 1) to 33% (pilot 2) said they would recommend the app to a friend. The usability scores measured by SUS (pilot 1: 73%; pilot 2: 64%) also followed this trend in which pilot 2 SUS scores were less than acceptable. In spite of this, 59% (pilot 1) to 50% (pilot 2) continued to use the app in the 8 weeks to some degree following the intervention. These data suggest the app may have been very useful for some, but not useful for others. Most participants (pilot 1: 83%; pilot 2: 86%) found the Facebook group helpful suggesting it added value to the intervention. A larger trial will be needed to compare how the app is used by individuals and how different use patterns/choices affect (1) user experience, (2) how much an individual finds the app helpful, and (3) how likely they are to recommend the app to others.

Participants lost weight during the 8-week intervention, which when converted to mean weekly weight loss (pilot 1: –0.20%; pilot 2: –0.30%), is fairly consistent with the weekly rate of weight loss over 6 months in the Diabetes Prevention Program (0.28%) [32]. However, further research is needed to determine whether weight loss from a program of this nature would continue through 6 months. In the 8 weeks after the intervention program ended, some weight regain was observed. Weight change in this intervention was highly variable with 37% (pilot 1) to 50% (pilot 2) losing 3% or more of their baseline weight in 8 weeks, but 38% to 41% not losing any weight. The Diabetes Prevention Program weight loss goal for 6 months was 7%, thus those achieving 3% or more over 2 months (8 weeks) were on track toward that goal. The lack of benefit for some participants suggests that remotely delivered weight loss programs may not be suitable for everyone. The sample was too small to explore predictors of intervention efficacy, but this will be an important question for larger trials.

Contrary to our hypotheses, no changes were observed in problem-solving skills. This may have been due to lack of power or it may be that having technology facilitate problem solving does not translate into improved problem-solving skills in the same way that human-delivered problem-solving therapy does. Only one of the three existing studies testing technology-delivered problem solving for depression examined problem-solving skills as an outcome and it did not find an effect [17]. In human-delivered problem-solving therapy, the therapist provides input into the selection of both problems and solutions. Efficacy of an app then would depend on the extent to which participants chose to work on problems that were significantly obstructing their weight loss progress versus problems that even if solved would not have much impact on weight loss. Patients might not always be aware of which problems if solved would deliver the best return on investment in terms of their weight. For example, one participant said she would have liked the app to help her increase her water consumption, a problem she felt was obstructing her weight loss. Because this problem was not in the app, she hand entered drinking more water as a solution so she could set reminders to do it. Unfortunately, the evidence for increasing water consumption on weight loss is weak [33], so using the app to build this habit would not likely result in much if any weight loss, especially to the extent that her energy was focused on this habit to the exclusion of others that would more directly impact energy balance. On the one hand, allowing participants to add their own solutions engages them in the brainstorming process; on the other hand, it may lead them to enter solutions that are ineffective. Although the counselor in the Facebook group can provide feedback to participants on the habits they choose, not all participants discussed the habits they chose in the group. Given the volume of misconceptions around weight management in public and professional discourse [34], some effort may be needed to debunk myths and keep patients focused on behavioral strategies that are supported by evidence.

Problem-solving skills may not have improved because the degree of automation of the process of problem solving compromised participant’s ability to learn the skills. The Habit app could be improved by including a way for the counselor to provide input into the problems and solutions selected by participants. The next iteration of the app will give the user feedback on whether the habits selected are resulting in weight loss and prompt them to switch habits when weight is not declining.

Automating the problem-solving process provides a unique opportunity to access data on specific habits and associated weight loss, which could lend insights into which specific habits work best for whom. In future work using mobile app-delivered problem solving, the specific problem and solution sets associated with the greatest weight loss should be identified so that users can be pointed to the habits that are likely to produce the greatest results. For example, habits could have an efficacy rating to let users know which have worked best for users like them. This would help streamline the number of solutions offered and perhaps curb people from adding solutions that have intuitive appeal but are not useful. Such data would also push our knowledge of which specific behavior changes are most impactful when it comes to weight loss. Behavioral weight loss interventions include a collection of strategies focused on myriad behaviors (eg, meal planning, shopping habits, exercise) that can be overwhelming for patients to implement all at once. Granular behavioral data collected from mobile apps provide an enormous opportunity to increase our understanding of behavior.

This study has a number of limitations. The sample sizes in both pilots were not powered to test efficacy on weight loss. Instead, the first step in this line of research was to examine the use and acceptability of the app to test “proof of concept” and inform refinements without the investment of a fully powered trial. Resources to retrieve clickstream data from the app were lacking and thus self-report was relied on to measure use, which may have been biased toward overreporting. Clickstream data would have provided data on the specific problems and solutions selected, although with such small samples conclusions drawn from these data would be very limited. Going forward, these data will be critical to answer questions about how participants use the app and how certain patterns of use are related to weight loss. Another limitation is the lack of diversity in the sample, a problem that has plagued weight loss intervention studies for decades [35]. Research investigating what men and non-white adults want from a weight loss mobile app is needed. We developed the app for the Android platform because Android was one of the two mobile platforms with the largest market share in the United States and availability of low-cost Android devices made this platform more accessible to diverse user groups compared to the iOS platform. Future research will include development of the app for iOS platform to increase eventual reach. Finally, the impact of the Facebook group and the Habit app cannot be disentangled; however, research shows that apps with no accompanying support are insufficient to produce weight loss.

The next step in this research is further developmental work and feasibility testing to (1) improve the impact of the app on problem-solving skills, (2) assist users in selecting problem and solution sets that have a high likelihood of impacting weight, and (3) better integrate the app (and problem-solving process) with the online social network. Mobile delivery of complex behavioral strategies may require extensive developmental work to achieve treatment fidelity and affect treatment mechanisms. For this reason, treatment fidelity and mechanisms should always be measured in pilot and feasibility studies of mobile apps that deliver behavioral strategies. Problem solving is among a collection of behavioral counseling strategies that if effectively and inexpensively implemented in the mobile environment could increase the reach and impact of behavioral interventions.

## Abbreviations

BMI: body mass index
SPSI-R: Social Problem-Solving Inventory-Revised
SUS: System Usability Scale
